# Tucker’s Reconstructive Laryngectomy: Indications and Functional Outcomes

**DOI:** 10.22038/ijorl.2024.78978.3660

**Published:** 2025

**Authors:** Malika El Omri, Wassim Kermani, Souhir Chelly, Mouna Bellakhdher, Mohamed Abdelkefi

**Affiliations:** 1 *Department of Ear, Nose, Throat and Head and Neck Surgery, Farhat Hached University Hospital, University of Sousse, Sousse, 4000, Tunisia.*; 2 *Infection Prevention and Control department, University Hospital Centre Farhat Hached, University of Sousse, Sousse, 4000, Tunisia.*

**Keywords:** Laryngectomy, Reconstruction, Glottis, Neoplasm, Treatment outcome

## Abstract

**Introduction:**

Frontal anterior laryngectomy with epiglottic reconstruction (Tucker’s reconstructive surgery) is a technique of partial laryngectomy that has been used by several authors since its introduction in the 80s.The aim of this serie is to specify the indications of this operation and to present the functional and oncological outcomes of our study and those found in the literature.

**Materials and Methods:**

We report a retrospective study of 65 cases who underwent Tucker’s operation by many surgeons at our educational center over a period of 31 years (1988 - 2020).

**Results:**

This serie included 62 men (95%) and 3 women (5%), with a mean age of 62,8 years. The cases were classified as follows: 42 patients with T1aN0M0 (65%), 21 patients with T1bN0M0 (32%) and 2 patients with T2N0M0 (3%). Following surgery, the mean time for tracheal cannula extraction was 18,4 days and for nasogastric tube was 14,4 days. Five cases developed aspiration pneumonia. Voice quality was then assessed based on its intensity and intelligibility with 11,7% rated as very good, 53,3% as good, 31,7% as average and 3,3% as poor. There were 4 cases of local recurrence, 2 cases of nodal recurrence, and 2 cases of tumour pursuit. The median survival rate was 7,5 years.

**Conclusion:**

The functional and oncological outcomes of Tucker´s reconstructive surgery were generally satisfactory in our patients and are consistent with those reported in the literature. This technique holds an important position of this technique in the therapeutic arsenal for early glottic carcinoma.

## Introduction

Glottic carcinoma is a frequent malignant tumour of the head and neck, with squamous cell carcinomas accounting for over 95% of laryngeal tumours. Early-stage glottic carcinoma (Tis, T1, and selected T2 cases) can be treated with partial laryngeal operation, transoral laser microresection or radiotherapy (1). The primary goals of partial laryngeal surgery are to manage the cancer surgically and preserve organ function for breathing, speech, and swallowing (2). In 1959, Majer et al. reported the supracricoid partial laryngectomy with cricohyoidoepiglottopexy (SCPL-CHEP). Later, in 1979, Tucker et al. advanced the reconstructive anterior frontal laryngectomy (RAFL) by modifying a procedure previously reported by Sedlacek and other European authors (3,4). This technique has been practiced in our Ear, Nose and Throat Department. This study analyses the indications and results of the technique and compares them with those of other studies. 

## Materials and Methods

A retrospective study was conducted in our department from 1 January 1988 to 31 December 2020, which included 65 patients who underwent Tucker’s operation. 

All cases had undergone surgery for glottic carcinoma classified as T1N0M0 (T: Tumour, N: Node, M: Metastases) or T2N0M0 according to the Union Internation

le Contre le Cancer (UICC) 2017. Cases underwent a RAFL procedure (T1a: The tumour is only in the left or right vocal cord, T1b: the tumour is in both vocal cords, T2: The tumour has reached the supraglottis. It may also affect the movement of the vocal cords, N0: There are no regional lymphadenopathy and M0: there are no distant metastases). 

The present study excluded patients with less than 12 months of follow-up or incomplete medical records, patients with tumours of subglottic, transglottic or supraglottic origin originating from the anterior commissure, those with a concurrent second primary, and with tumors not amenable to partial laryngectomy for oncological and/or functional reasons. None of the patients included in the study had underwent previous surgical treatment or received radiotherapy. 

The patients´ caracteristics including age, sex, smoking history and alcohol consumption were recorded. The absence or presence of dysphonia, laryngeal dyspnea, dysphagia, or otalgia was also noted. The initial location of the tumour, the mobility of vocal cords, and any metastases were determined through physical examination, endoscopy, and laryngeal tomography: thin sections are more important than in computed Tomography (CT) scan and but it still provide a clear view of the laryngeal airway.

The tumour was staged conforming to the TNM classification (UICC- 2017) based on these findings.

The surgical technique performed in all patients was RAFL, which involves the following steps: performing tracheotomy and separating the muscles below the hyoid bone to expose the thyroid cartilage. Two perichondrial flaps were obtained by incising the perichondrium of the thyroid cartilage in the midline. Two vertical parallel incisions were made in the thyroid wing. Scissors were used to cut the less involved side of the cartilage. The excision requires the anterior two-thirds of the thyroid cartilage, but may include the total vocal cord up to the arytenoid, separating the anterior two-thirds of the ipsilateral cord. 

After performing a trans-epiglottic horizontal laryngotomy, the surgeon dissects the vocal cord ligament, ventricular band, and thyroarytenoid muscle anterior to the vocal process to the cricoid cartilage. 

**Fig 1:**
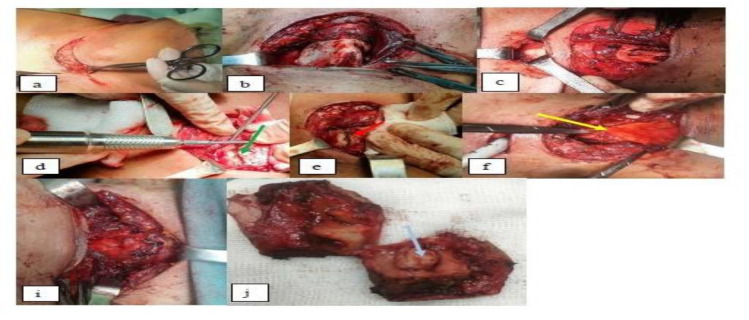
Main stages of LFAR: a) Skin incision opposite the crico-thyroid membrane, b) Exposure of the thyroid cartilage, c) Tracheotomy, d+e) Thyroid cartilage incised vertically on the midline and roughened up to the junction of the posterior 1/3 and anterior 2/3 of the thyroid wings( ), f) Epiglottoplasty : The epiglottis is easily lowered by traction downwards to the upper edge of the cricoid after removal of the surgical specimen ( ), i) :Closure: the two flaps of perichondrium are sutured, then the subhyoid muscles are brought together by several stitches, j) Surgical specimen showing a tumour occupying the entire right vocal cord ( ) .

The tumour is then resected under direct vision. The epiglottis is repositioned to the edge of the thyroid cartilage, and its inferior edge was fixed to the cricoid cartilage. The infrahyoid muscles above are sutured together without tension (Figure 1). 

Data was collected on early complications of surgery, including sepsis, haematoma and aspiration pneumonia. We also recorded the delay in decannulation, the delay in the first oral feeding, the time of tube removal, and the length of hospital stays.

After speech rehabilitation, the orthophonist judged the postoperative voice quality based on its intensity and intelligibility, rating it as very good, good, fair or poor.

We also noted any local pursuit and recurrence and calculated overall survival. 

## Results

Between 1988 and 2020, our department admitted 65 patients with glottis carcinoma. Of these 62 were men (95%) and 3 were women (5%) with a sex ratio of 0,05. The patients´ age ranged from 32 to 75 years, with a mean age of 62,8 years. Only 6 patients (9%) reported no history of smoking or alcohol consumption. Fifty-four patients reported smoking and 39 (60%) had a history of combined smoking and alcohol abuse. All cases had dysphonia, with one patient experiencing laryngeal dyspnea.None of the patients reported odynophagia or otalgia. Prior to surgery, we assessed the initial tumour location, invasion and glottic mobility through indirect and direct laryngoscopy. Cervical examination was used to assess lymph node involvement. CT scan was performed in 50 cases and laryngeal tomography in 15 cases and all cases were squamous cell carcinoma. Data on tumour location and tumor extension are summarised in table 1(Table 1). 

**Table 1:** Tumor parameters

**Tumour extension**		**Number of cases**	
True vocal cord	With anterior commissure	28	30
	Without anterior commissure	2	
Anterior two-thirds true vocal cord	Anterior third of the contralateral true vocal cord	1	8
	Anterior commissure	5	
	Dyplasia of the contralateral true vocal cord	2	
Anterior third of true vocal cord and anterior commissure		4	
Anterior third of two true vocal cord and anterior commissure		7	
True vocal cord and the anterior third of the contralateral true vocal cord		12	
Anterior commissure		2	
Anterior third of two true vocal cord and anterior commissure and discrete subglottic extension		2	

Patients were classified as follows: the study included 42 patients with T1aN0M0 (65%), 21 patients with T1bN0M0 (32%) and 2 patients with T2N0M0 (3%). Arytenoid cartilage resection was performed in 31 cases (47,7%). Three ipsilateral neck dissections of level II, III, and IV were performed due a slightly overflowing lesion on the floor of the laryngeal ventricle. There were no lymph node metastases in the final histological result.Postoperative histopathological examination revealed that 64 patients (98,5%) had a tumour-free resection margin, while one patient had a positive surgical resection margin. 

In 12 cases (18,5%) we found no involvement of the Delphien node based on the frozen section diagnosis. The immediate postoperative results are summarized in table 2, which shows no surgical deaths (Table 2).

**Table 2:** Functional parameters

**Immediate postoperative outcome**	**Number of days**
Decannulation	18,4 (10-44 days)
First oral feeding	10,3 (4-16 days)
Nasogastric tube removal	14,4 (7-39 days)
Hospitalstay	23 (14-60 days)

Three diabetic patients suffered a heart stroke postoperatively, two of whom were treated successfully, while one patient died. There were no cases of death resulting from aspiration pneumonia or chronic aspirations.

Speech and swallowing rehabilitation were initiated on day 7. The first oral nourishing tests began between 7^th^ and 20^th^ days and the median delay was13 days. The diet began gradually with pasty, then semi-liquid and finally liquid. The nasogastric tube was removed after the patient tolerate liquid food without aspiration while swallowing. Decannualtion was performed after the airway was appropriate and the patient did not have aspiration while swallowing. Voice quality was evaluated based on its intensity and intelligibility. The results showed that 11,7 % of cases had a very good voice quality, 53,3% had a good voice quality, 31,7% had a fair voice quality and 3,3% of cases had a poor voice quality.Pneumonia due to aspiration was observed in 5 cases, but they were successfully treated with antibiotics. Additionally, 8 patients (12,3%) had chronic aspiration, with 7 cases (10,7%) resulting from arytenoid cartilage resection.The results for cancer were as follows: The average recurrence ranged from 2 to 20 years, and the average survival was 7,5 years. Early recurrence (<3 months) was observed in only 2 cases (3%), one of which had insufficient resection margins. One patient underwent total laryngectomy and bilateral functional neck dissections followed by radiotherapy, while the other patient refused surgery and subsequently passed away. Four patients (6%) experienced late recurrence, with time to recurrence ranging from one to fifteen years. Of these, four cases underwent total laryngectomy and bilateral functional neck dissections, while two received adjuvant radiotherapy. One patient died before receiving radiotherapy and the other was lost to follow-up. Lymph node recurrence was observed in 2 cases, occurring at one year and three months respectively. The initial patient had a large subclavicular node and received palliative chemotherapy, while the second patient underwent radical neck dissection and external radiation therapy. These two patients had no prior medical history. They experienced lymph node recurrence despite sufficient tumour margins during partial laryngectomy. 

**Fig 2:**
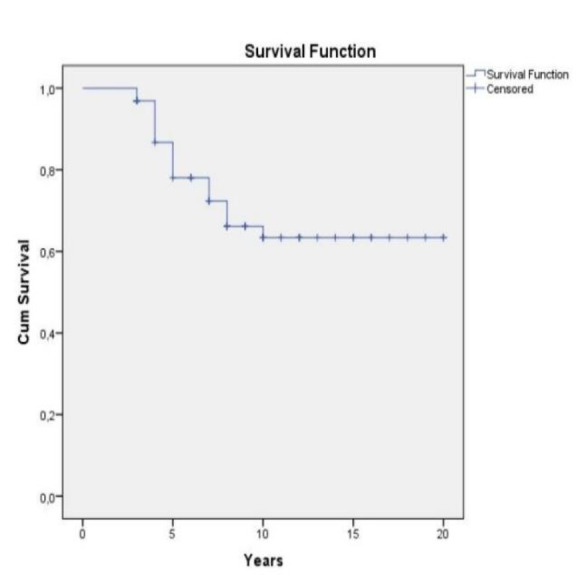
Overall survival curve

The overall survival rates were 96.9% at 3 years, 78% at 5 years and 63,4% at 10 years. The disease free-survival at 5 years was 67,7%. (Figure 2). Patients with a poor outcome with this surgical technique had higher TNM scores, indicating that the surgical technique was not optimal.

## Discussion

This study found that patients who underwent RAFL surgery had stage T1 and T2N0M0. All patients complained of dysphonia, which was the most common symptom leading to diagnosis. Physical examination can determine tumor extension and vocal cord mobility, which is crucial for establishing indications and contraindications for this technique. 

Some authors propose that the indications for RAFL include (3,5,6):

Unilateral carcinoma of the true vocal fold that extends to the superficial layer of the anterior commissure and the vocal process of the arytenoid or to within 0.5 cm under the subglottis. Bilateral tumours of the true vocal fold are acceptable, without or with involvement of an arytenoid, as long as the tumour does not extend into the ipsilateral vocal fold by more than 50% of the vocal cord.Superficial and small tumours that are localized in the transglottic area and involve the lower part of the ipsilateral recurrent vocal fold and the ventricle are suitable for RAFL. 

However, there are certain contraindications to this procedure including: epiglottis, subglottic spread of the tumour more than 0,5 cm laterally and 1 cm anteriorly, immobility of the true vocal cords due to tumour invasion, thyroid cartilage infiltration, severe lung disease, and age over 75 years (3,5). 

The purpose of a cervical CT scan is to define the spread of tumour invasion into the peri epiglottic space, anterior to the anterior commissure, thyroid cartilage, lateral to the paraglottic space and cervical lymph nodes. This evaluation can assist in selecting the most appropriate laryngectomy technique. It is important to note that important extent to the anterior commissure or extent to ventricular ligaments and the ventricles contraindicate this procedure (3,6,7). 

 In our study, we conducted cervical CT scans in 50 cases and laryngeal tomography in the remaining cases as they are old cases. It´s important to note that thyroid cartilage infiltration is also a contraindication to this technique. Therefore, magnetic resonance imaging (MRI) is increasingly being performed as a qualified imaging procedure for the detection of tumour involvement of the thyroid cartilage (8,9). However, in our series, MRI was not performed. 

Regarding the postoperative outcome, the duration of hospital stay was almost identical to that reported in published studies. The average time to decannulation in our study was 18,4 days, which is consistent with other studies. In Mallet´s study, decannulation was performed after satisfactory oral feeding, usually around two weeks after the operation (6). In Lawson and Yagiz, the cannula was removed as soon as the absence of a false passage was demonstrated on swallow x-ray (4,10).

In our study, the delay for oral feeding was 10,3 days and the tube was removed after 14,4 days. Mallet recommended shorter periods for nasogastric tube ablation and the mean time for removal was about 12 days (6), Oysu recommended longer periods, and nasogastric tube was removed after an average of 17 days (11). Lawson estimated the incidence of aspiration pneumonia to be about 9% (4). 

In evaluating patients´voice, the Voice Handicap Index (VHI) questionnaire was used by authors, which was proposed by Jacobson (12). The questionnaire appreciates the impact of voice dysfunction on cases' quality of life in 3 aspects: Functional (F), Emotional (E) and Physical (P) and with an overall score as Global (G) (13). In our study, we did not use the VHI questionnaire. Instead, we judged voice quality was based on its intensity and intelligibility. In our study, we did not use the VHI questionnaire. Instead, we judged voice quality was based on its intensity and intelligibility. 

The literature report a hospital stay of less than 22 days after RAFL, which is consistent with our study where the average hospital stay was 23 days (4,6). 

Complications following RAFL include rupture of the base of the epiglottis, posterior ptosis of the epiglottis (which may require tracheotomy), aspiration pneumonia and laryngeal stenosis (3,4,6). Our study found that 5 cases developed aspiration pneumonia, which was treated with antibiotics, and 8 patients developed chronic aspiration. 

The optimal treatment for glottic cancer is a matter of debate. Glottic tumour can be treated with either external radiotherapy or partial laryngectomy (transoral endoscopic resection or open surgical resection). Both surgery and radiotherapy have been shown to be effective in treating similar cancers. Radiotherapy is often the favored primary therapeutic option for patients with a higher demand for voice quality (14). Patients with significant medical comorbidities, who are not suitable candidates for anaesthesia, may also benefit from radiotherapy (15). 

However, radiotherapy is a time-consuming way and is associated with long-term oncological problems such as chondronecrosis and oedema, as well as hidden costs (16).

The reported failure rates for T1 and T2 glottic lesions with primary radiotherapy are 5% to 10% and 20% to 40%, respectively (14,16). 

Total laryngectomy is often performed in cases where achieving negative tumour margins in a fibrotic oedematous larynx, difficult as irradiated cartilage increases the risk of complications such as necrosis and local infection (16). 

Endoscopic carbon dioxide (CO2) laser surgery or transoral CO2 laser microsurgery has emerged as an effective treatment for early laryngeal cancer due to its good functional and oncological results (11). Frontal anterior laryngectomy is an extended type VI resection (17) that involves the removal of the epiglottic stalk, the anterior part of the ventricles and the vocal cords, the anterior commissure, and the nearer subglottic mucosa (17,18).

Transoral CO2 laser microsurgery has several advantages over radiotherapy and partial surgery, including shorter treatment and hospital stays. Endoscopic resection offers the advantage of being repeatable in case of recurrence (15) and allows for reserving radiotherapy for second primary lesions or recurrences. Unlike radiotherapy and open conservative laryngectomy, endoscopic resection does not require tracheotomy (16). 

A contraindication to endoscopic resection is still considered by some authors to be tumour involvement of the anterior commissure (11).

Transoral laser resection may be impossible in certain cases due to patient morphology, comorbidities, or the non-availability of a laser or a skilled surgeon in the procedure, transoral laser resection may be not possible (11,19). 

The voice quality is similar to that after radiotherapy and comparable to that after external partial surgery, depending on the type of operation performed (20).

According to the literature, chemotherapy alone does not provide any advantage over conservative laryngeal surgery or radiotherapy as a therapeutic alternative. However, the evolution of biological markers of chemosensitivity may enable the choice of cases who can benefit from chemotherapy alone in the future (21). 

Various procedures have been reported for the open surgery of T1-T2 glottic carcinoma. These techniques can be broadly classified into vertical partial laryngectomies, including vertical thyrotomy with open cordectomy and anterolateral vertical partial laryngectomies, vertical hemi-laryngectomy and anterior vertical partial laryngectomies, vertical hemi-laryngectomies, frontal anterior vertical partial laryngectomy, and horizontal partial laryngectomies, mainly defined by the family of SCPL-CHEP, cricohyoidopexy, or tracheo cricohyoidoepi- glottopexy (17). 

Local recurrence or persistence was detected using indirect laryngoscopy. In cases of doubt, endoscopy with biopsy and microscopic examination was performed. The literature suggests that an inadequate resection of tumour margins is the most significant factor for recurrence (3). In our study, only one patient had insufficient tumour margin out of the 6 cases (9%) of recurrence. 

Predictive factors for lymph node recurrence include insufficient resection of tumour margins and local recurrence, as stated in the literature. However, in our study, two patients experienced lymph node recurrence despite sufficient tumour margins during partial laryngectomy. Therefore, clinical follow-up is necessary for early detection of recurrence, assessment of functional parameters and accelerated support (3,10,21). 

## Conclusion

The functional and carcinological outcomes of frontal anterior laryngectomy with epiglottis reconstruction were satisfactory in our patients and are consistent with those reported in the literature, indicating that this technique hold a significant place in the therapeutic options for early glottic cancers. 

Our study analysed the functional and carcinological outcomes of this partial surgery.Adequate management of post-operative complications enable patients to heal rapidly, facilitates early decannulation, and promises the early recovery of physiological functions.
